# Rudimentary horn pregnancy in the first trimester; importance of ultrasound and clinical suspicion in early diagnosis: A case report

**DOI:** 10.4274/tjod.10437

**Published:** 2014-09-15

**Authors:** Hasan Terzi, Arzu Yavuz, Ömer Demirtaş, Ahmet Kale

**Affiliations:** 1 Kocaeli Derince Education and Research Hospital, Clinic of Obstetrics and Gynecology, Kocaeli, Turkey; 2 Pamukkale University Faculty of Medicine, Department of Obstetrics and Gynecology, Denizli, Turkey

**Keywords:** Preoperative diagnosis, rudimentary horn pregnancy, ultrasonography

## Abstract

We aimed to present 7-8 weeks rudimentary horn pregnancy detected preoperatively. A 37-year-old woman, gravida 3, para 2, at 7-8 weeks’ gestation referred to our clinic with a complaint of abdominal pain. The patient was primarily infertile, and she had unicornuate uterus detected during infertility investigation. Due to abnormal ultrasonographic image, rudimentary horn pregnancy was considered. Accurate diagnosis was made by laparoscopy, and rudimentary horn excision was performed. Prerupture diagnosis is very difficult in rudimentary horn pregnancies. The key role in preoperative diagnosis is suspicion. Ultrasonographic examination and clinical suspicion are sufficient in most cases.

## INTRODUCTION

The female genital system includes the uterus, the cervix, fallopian tubes and upper third of the vagina, all formed from the mullerian ducts during the 7^th^ week of gestation. Anomalies during the fusion of the mullerian ducts are the basis of rudimentary horn. Because most cases are asymptomatic, the incidence is not well known, and most rudimentary horn cases exist generally in benign form. Only cases with endometrial cavity are noticed if dysmenorrhea and chronic pelvic pain occurs during puberty or if rupture and intraabdominal hemorrhage occur during a rudimentary horn pregnancy. Suspicion plays a key role in the diagnosis of these cases. In order to decrease complications during rudimentary horn pregnancy, preoperative diagnosis services need to be usedappropriately and timely. Even though recent medical developments have made the diagnosis of the rudimentary horn pregnancy easier, lack of experience in the diagnosis criteria and symptomatology are the most predominant issues that affect the rise of complications during rudimentary horn pregnancy. This paper presents the techniques and criteria used during preoperative diagnosis of a rudimentary horn pregnancy. As a result of this diagnosis, it is shown that minimally invasive techniques can be used for treatment.

## CASE

**Figure 1:**
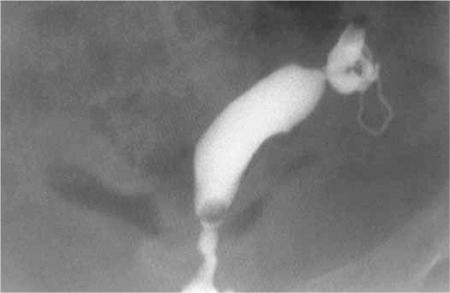
The view obtained from an HSG (from 8 years ago) showing unicornuate uterus during the infertility investigation

A 37-year-old, gravida 3 and parity 2 patient with abdominal pain was admitted to the hospital. The anamnesis revealed primary infertility, and unikornuate uterus in a previous hysterosalpingography (HSG) (Figure 1). Both of the patient’s pregnancies were spontaneous.

**Figure 2:**
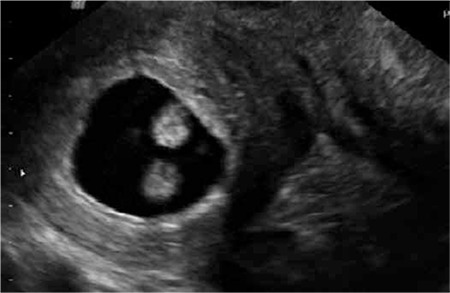
A thick myometrial tissue (separate from the uterus) surrounding the gestational sac and embryo during the transvaginal ultrasound study

**Figure 3:**
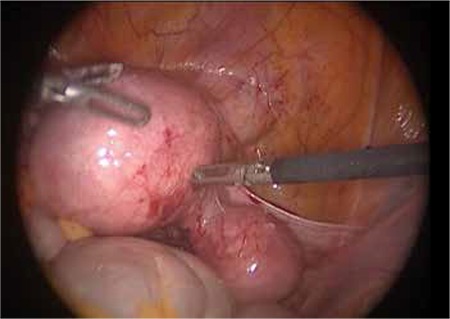
The laparoscopic view the rudimentary horn pregnancy in the right adnex

**Figure 4:**
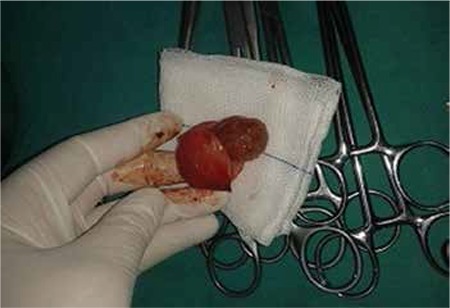
Laparoscopically removed gestational sac inside the rudimentary horn

When admitted to the hospital, the patient’s vitals were stable. The patient’s arterial blood pressure was measured as 120/70 mmHg and her pulse rate was measured as 78 bpm. Palpation examination of the abdomen showed tenderness with no observed defense or rebound. Gynecological examination showed the perineum, vagina and vulva were normal and collum forme as closed. No vaginal hemorrhage was observed. Uterus was at an 8-week size, left adnex was nonpalpable and a mobile mass with a diameter of 5 to 6 cm was palpated on the right adnex. Transvaginal ultrasonography showed an adnexal mass having an embryo with an observed fetal heart rate, a gestational sac of 30x27 mm, and a crown-rump-length (CRL) of 12.4 mm corresponding to pregnancy of 7 weeks 3 days (Figure 2). The uterine cavity had no visible gestational sac. The pouch of douglas had no free fluid inside. Patient’s bloodwork showed a beta-hCG level of 30742 mlIU/ml, hemoglobin level of 12.5 g/dl and Htc level of 37.5%. From the above results, along with the anamnesis of primary infertility and unicornuate uterus, it was thought that the patient might have rudimentary horn pregnancy, and therefore, a laparoscopic surgery was planned. Laparoscopic pelvic anatomy showed the right ovary and tuba uterina, along with a noncommunicating rudimentary horn with a diameter of 5-6 cm, in the right adnexal region. Left ovary and tuba uterina were both normal, and uterus was unicornuate (Figure 3). A rudimentary horn excision was also performed. The rudimentary horn was pulled out of the abdomen with the help of an endobag extractor (Figure 4). In addition, a right salpingectomy was performed. Surgical operation concluded after a check for bleeding. The pathology report was consistent with rudimental uterus and pregnancy.

## DISCUSSION

Rudimentary horn is a mullerian anomaly that is a variant of unicornuate uterus. Rudimentary horn pregnancy is very rare with an incidence level between 1/76000 and 1/140000^(1)^. According to the American Society for Reproductive Medicine (ASRM) mullerian anomalies can be divided into 7 sub-groups, and unicornuate uterus is categorized into 4 groups^(2)^. ASRM indicates that the most common unicornuate uterus case is the horn with a fibrous joint to the unicornuate uterus without endometrial cavity. Most patients are asymptomatic and incidental. Rudimentary horn pregnancy is an ectopic pregnancy that exhibits pregnancy ruptures. It is easy to understand the pathophysiology when there is a communication between the rudimentary horn and the other uterus and servix. In the absence of this continuity, it is still possible to observe a horn pregnancy, and it is thought that the pregnancy is possible with sperm transperitoneal migration^(3,4)^. Due to previous hysterosalpingogram (HSG) results showing no contrast agents, it is thought that the pregnancy is related to the sperm transperitoneal migration in this case. In this case, corpus luteum was observed on the same side as the rudimentary horn.

Early diagnosis of rudimentary horn is imperative. Present day, preoperative diagnosis cases are constantly increasing, and diagnosis is usually made between the 6^th^ and 13^th^ weeks of pregnancy. The first trimester of pregnancy is the most important period for such a preoperative diagnosis. It becomes very difficult to diagnose a rudimentary horn pregnancy after the first trimester. The unnoticed rudimentary horn pregnancy inevitably ends with a rupture^(5)^. 80% to 90% of the cases have ruptures between the 10^th^ and 20^th^ weeks of pregnancy^(1,6)^.

Average rudimentary horn pregnancy is predicted to last about 21-22 weeks. Even though cases with longer pregnancies have been reported, cases almost always end with a rupture. The fetus reaches full term in 10% of the cases, and fetuses survive in only 2% of these cases^(7)^. With rudimentary horn pregnancies, results such as intrauterine growth restriction (IUGR), presentation anomalies and abnormal placentation can occur. The neonatal findings of full term babies, born through a rudimentary horn pregnancy, are also not very good.

Half of rudimentary horn pregnancies are diagnosed after the rupture. The diagnoses at later stages are cases where rudimentary horn pregnancy went unnoticed during the first trimester. The sensitivity of ultrasonography, which is the simplest diagnosis technique, in detecting such an anomaly is 26%^(8)^. Maternal mortality is currently at such a low level that it almost does not exist. The main reasons for the reduction in maternal mortality are developments in diagnosis techniques and improvements in diagnosis criteria, along with an increase in preoperative diagnosis cases. The following criteria have been suggested by Tsafrir et al for ultrasonographic diagnosis of rudimentary horn pregnancy^(9)^:

1. Pseudopattern of an asymmetrical bicornuate uterus.

2. Absence of visual continuity between the cervical canal and the lumen of the pregnant horn.

3. The presence of thick myometrial tissue surrounding the gestational sac.

In this case, due to the anamnesis of primary infertility and unicornuate uterus, the possibility of rudimentary horn existed. Therefore, it was thought that first trimester related studies needed to be performed. The investigation, based on the above criteria, revealed that the gestational sac was clearly unattached from the uterus, and a thick myometrial echo texture surrounded the gestational sac. Because the existing results showed a rudimentary horn pregnancy, we felt that magnetic resonance imaging (MRI) was not needed.

As mentioned before, suspicion is imperative in identifying the existence of rudimentary horn, which is a component of mullerian anomalies or rudimentary horn pregnancy. Even though ultrasonographic diagnosis success rates are low, conditions such as infertility, chronic pelvic pain and edometriosis should raise the attention of doctors in cases with anamnesis of unicornuate uterus or urinary tract anomalies. In such cases, pregnancies during the first trimester should be closely monitored^(10)^.

In cases with the presence of unicornuate uterus or with the anamnesis of unicornuate uterus, an investigation into the existence of rudimentary horn or urinary tract anomalies needs to be performed. Because the urinary system and mullarian system codevelop during the embryonic stage, urinary tract anomalies can also be seen. Of these urinary tract anomalies, renal agenesis and ectopic kidney are most frequently observed. MRI and intraveneous pyelogram (IVP) are imperative in the diagnosis of such cases. Excision, right after the diagnosis of rudimentary horn, is the most accepted treatment method today. Some authors have reported success with fetal intracardiac potassium choloride (KCl) injection and/or methotrexate treatment^(6)^. However, rudimentary horn should not be left in the body due to the possibility of ectopic pregnancy. Rudimentary horn excision can be done through laparotomy or laparoscopy. Most patients with preoperative diagnosis have been treated with laparascopy because it is much easier to remove connections and pull the rudimentary horn out of the abdomen during the early stages of pregnancy. The authors preferred laparoscopy in this case for the above mentioned reasons. During rudimentary horn excision, the tuba uterina should not be left since there is a case study in which the rudimentary horn was formed inside the tuba uterina on the side where rudimentary horn was first observed^(11)^. The authors performed tubal excision in this case, as well.

In conclusion, an increased level of experience, along with careful ultrasonography during the first trimester, is key to reducing rupture complications that result from rudimentary horn pregnancies. For this purpose, careful analysis of the patient’s ultrasonographic criteria and anamnesis may be enough for the diagnosis. Spreading awareness among doctors is an important step for increasing the chances of preoperative diagnosis of rudimentary horn pregnancies.
